# Leveraging logical definitions and lexical features to detect missing IS-A relations in biomedical terminologies

**DOI:** 10.1186/s13326-024-00309-y

**Published:** 2024-05-01

**Authors:** Rashmie Abeysinghe, Fengbo Zheng, Jay Shi, Samden D. Lhatoo, Licong Cui

**Affiliations:** 1 Department of Neurology, The University of Texas Health Science Center at Houston, Houston, TX USA; 2 McWilliams School of Biomedical Informatics, The University of Texas Health Science Center at Houston, Houston, TX USA; 3 Intermountain Healthcare, Denver, CO USA

**Keywords:** SNOMED CT, NCI thesaurus, Terminology quality assurance

## Abstract

Biomedical terminologies play a vital role in managing biomedical data. Missing IS-A relations in a biomedical terminology could be detrimental to its downstream usages. In this paper, we investigate an approach combining logical definitions and lexical features to discover missing IS-A relations in two biomedical terminologies: SNOMED CT and the National Cancer Institute (NCI) thesaurus. The method is applied to unrelated concept-pairs within non-lattice subgraphs: graph fragments within a terminology likely to contain various inconsistencies. Our approach first compares whether the logical definition of a concept is more general than  that of the other concept. Then, we check whether the lexical features of the concept are contained in those of the other concept. If both constraints are satisfied, we suggest a potentially missing IS-A relation between the two concepts. The method identified 982 potential missing IS-A relations for SNOMED CT and 100 for NCI thesaurus. In order to assess the efficacy of our approach, a random sample of results belonging to the “Clinical Findings” and “Procedure” subhierarchies of SNOMED CT and results belonging to the “Drug, Food, Chemical or Biomedical Material” subhierarchy of the NCI thesaurus were evaluated by domain experts. The evaluation results revealed that 118 out of 150 suggestions are valid for SNOMED CT and 17 out of 20 are valid for NCI thesaurus.

**Supplementary Information:**

The online version contains supplementary material available at 10.1186/s13326-024-00309-y.

## Introduction

Throughout the years, biomedical terminologies have played a significant role in biomedical research and applications, especially in facilitating data management. Two such leading biomedical terminologies are SNOMED CT and National Cancer Institute (NCI) thesaurus. SNOMED CT is the world’s largest clinical terminology, which is a standard for facilitating the exchange of clinical health information [1]. NCI thesaurus (NCIt) is a reference terminology that facilitates translational research in cancers [2].

Many modern biomedical terminologies including SNOMED CT and NCIt have been formally represented using description logics (DL), a family of formal knowledge representation languages. A key reasoning service provided by DL is ontology classification, achieved by DL reasoners (e.g., ELK [3], Snorocket [4]), which can check the consistency of definitions across the whole ontology and automatically infer a hierarchy of concepts (i.e., infer IS-A hierarchical relations among concepts) based on the stated facts.

**Fig. 1:**
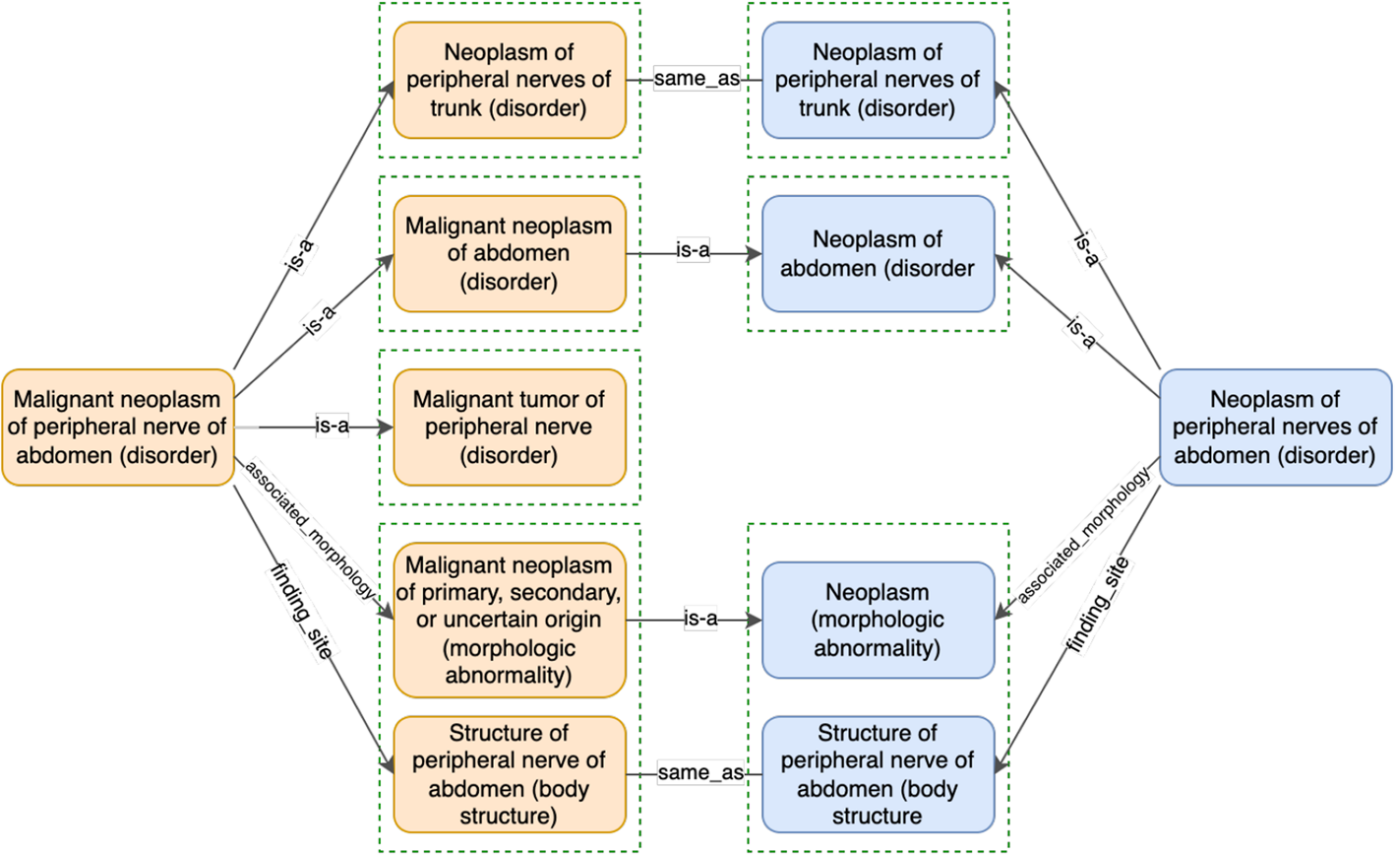
Comparison of inferred definitions of “*Malignant neoplasm of peripheral nerve of abdomen (disorder)*” (188326001) and “*Neoplasm of peripheral nerves of abdomen (disorder)*” (126992002) in the March 2020 Release of the SNOMED CT (US Edition) [10]. Relationship groups are indicated with dashed lines in green

In both SNOMED CT and NCIt, concepts are logically defined with hierarchical and attribute relations [5, 6]. The curators associate each concept with a stated definition consisting of description logic axioms based on the current knowledge about that concept. Then, a description logic classifier is applied to the stated definitions to generate inferred logical axioms [7]. For instance, Fig. 1 shows the inferred logical definitions of two SNOMED CT concepts: “*Malignant neoplasm of peripheral nerve of abdomen (disorder)*” and “*Neoplasm of peripheral nerves of abdomen (disorder)*”. In SNOMED CT, some relations are grouped into relation groups if they are associated with each other [8].

In SNOMED CT and NCIt, a concept is considered to be fully defined if its definition is sufficient to distinguish its meaning from other similar concepts [6, 9]. Otherwise, its definition status is primitive. If concept *A* is fully defined, the DL reasoners will identify concepts whose definitions satisfy *A*’s defining relations (i.e., whose definitions are more detailed/specific) to be the subtypes of concept *A*. On the other hand, if a concept is primitive, the DL reasoners will not infer any subtypes for it.

**Fig. 2:**
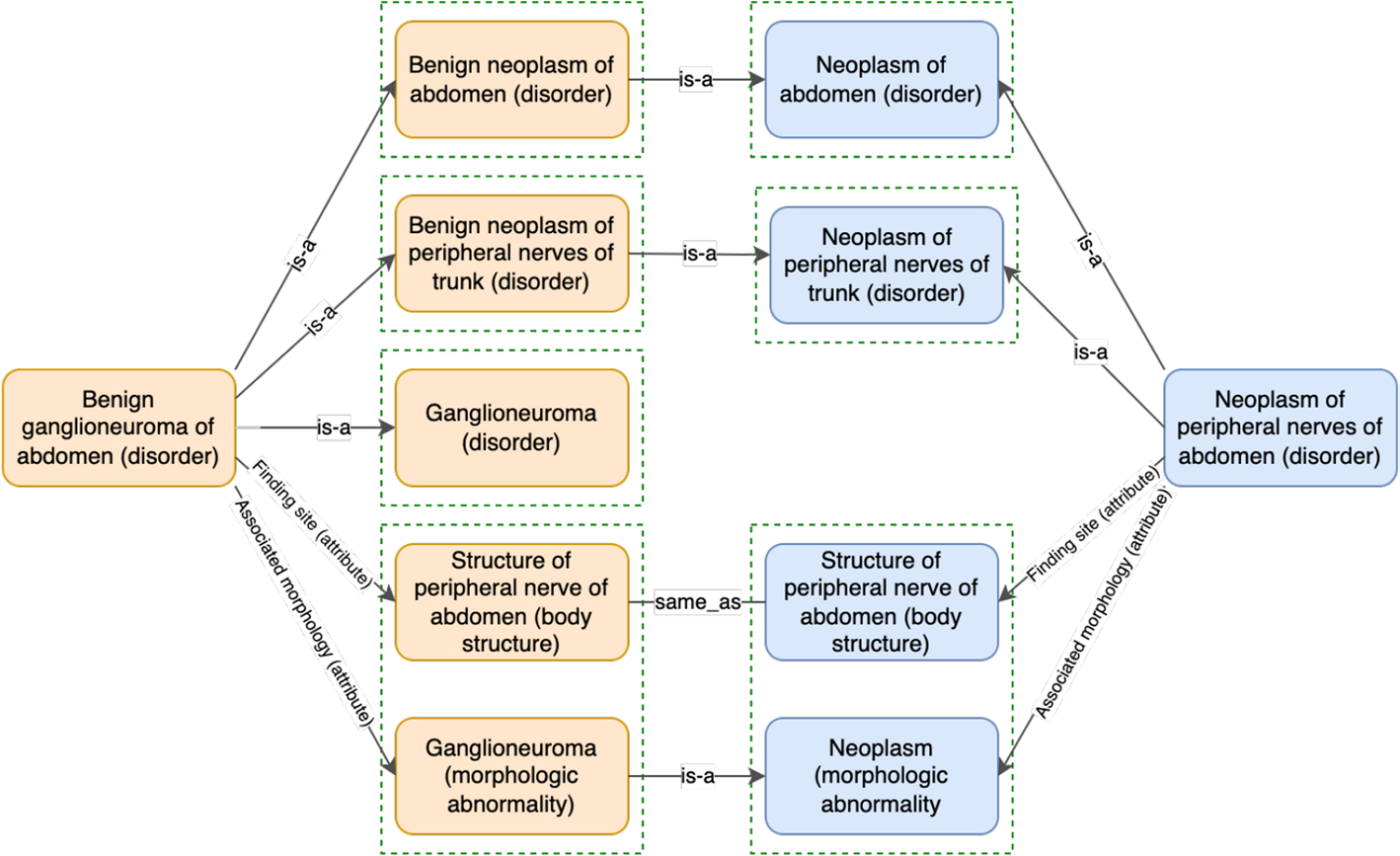
Comparison of inferred definitions of “*Benign ganglioneuroma of abdomen (disorder)*” (426134002) and “*Neoplasm of peripheral nerves of abdomen (disorder)*” (126992002) in the March 2020 Release of the SNOMED CT (US Edition) [10]. Relationship groups are indicated with dashed lines in green

The definition status (i.e. fully defined or primitive) of individual concepts is usually decided by the curators of the terminology. Therefore, valid hierarchical relations among concepts may not be captured by the DL reasoners due to the primitive definition status of the potential supertypes. For instance, in the March 2020 release of the SNOMED CT (US Edition), the concept “*Neoplasm of peripheral nerves of abdomen (disorder)*” is a primitive concept. As shown in Fig. 2, the definition of the concept “*Benign ganglioneuroma of abdomen (disorder)*” is more specific than this concept. This is because the corresponding attribute-value pairs (that are shown on the same level) are either the same or more specific in “*Benign ganglioneuroma of abdomen (disorder)*”. Similarly, as shown in Fig. 1, the concept “*Neoplasm of peripheral nerves of abdomen (disorder)*” is a primitive concept, and the definition of the concept “*Malignant neoplasm of peripheral nerve of abdomen (disorder)*” is more specific than this. However, still, DL reasoners will not establish a hierarchical relation between these two concepts (i.e., a missing hierarchical relation) as “*Neoplasm of peripheral nerves of abdomen (disorder)*” is primitive. Note that in the March 2021 Release of the SNOMED CT (US Edition) this hierarchical relation exists as “*Neoplasm of peripheral nerves of abdomen (disorder)*” became fully defined and hence the relation became derivable by DL reasoners.

Our goal in this paper is to identify such potentially missing hierarchical relations that the DL reasoners missed (i.e., in which the potential subconcepts are more specific than the superconcepts in terms of logical definitions, but the superconcepts are primitive). To achieve this, we first identify candidate pairs of concepts from non-lattice subgraphs which often contain quality issues including missing hierarchical relations. Then, given a candidate pair, we check if the inferred logical definition of one concept is more specific than that of the other. If so, the potential superconcept should be a primitive concept (otherwise, the hierarchical relation should have been inferred by the DL reasoners) and, there may be a missing hierarchical relation between these two concepts. Since the superconcept is of primitive definition status (i.e., the logical definition may be insufficient to express its semantic meanings), purely relying on the logical definition may lead to erroneous missing hierarchical relations being suggested. Therefore, in this paper, we also utilize lexical features of concepts as supplementary to determine the subsumption relations among concepts.

Throughout the years, there has been considerable exploration of various approaches to identify and address different quality issues including missing hierarchical (IS-A) relations within biomedical terminologies [11]. For instance, Bodenreider has come up with an approach to generate logical definitions of SNOMED CT concepts by lexical features in concept labels. Reasoning on these logical definitions has revealed missing hierarchical relations in SNOMED CT [12]. Graph summarization techniques (called abstraction networks) have been extensively utilized to uncover various modeling issues within biomedical terminologies [13–16]. Abstraction networks summarize the terminology structure and various characteristics of such networks have been investigated to address different quality issues. Agrawal et al. have explored different approaches to identify concepts that are lexically similar and should be modeled in a similar manner. Inconsistent modeling among such groups of concepts has led to the identification of errors [17–19]. Liu et al. have explored deep learning to suggest missing IS-A relations in NCIt [20, 21]. Their strategy involves training a Convolutional Neural Network with existing relations as positive samples and uncle-nephew pairs as negative samples. Concept features to train the model are obtained through documents containing concept lexical and hierarchical information. In previous work, we investigated training a Graph Neural Network to predict missing IS-A relations within the Clinical findings subhierarchy of SNOMED CT [22]. We utilized four types of features to train the model: concept name features; hierarchical features; enriched lexical attribute features; and logical definition features. A cross-validation-inspired approach was used to apply the model to all hierarchically unrelated concept pairs. In previous work, we have also proposed several approaches that uncover missing IS-A relations purely utilizing lexical features of concepts [23–29], and approaches that combine lexical and structural features [23, 24, 30]. A more detailed comparison with such approaches that are related to this work is provided later in the paper in the Discussion section.

## Methods

There are mainly four steps in our method: (1) pre-compute non-lattice subgraphs and identify candidate pairs of concepts that are currently not linked by hierarchical relations; (2) given a candidate pair, check if the inferred definition of one concept is more specific than the other’s; (3) compute lexical features for concepts and perform lexical-based subsumption checking; and (4) remove redundant and cycle-causing potentially missing hierarchical relations.

### Pre-computing non-lattice subgraphs and generating candidate pairs

**Fig. 3:**
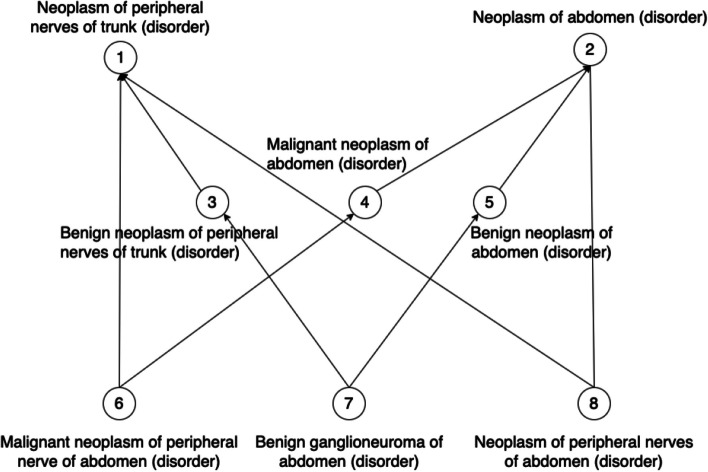
An example of non-lattice subgraphs in the March 2020 Release of the SNOMED CT (US Edition). Concepts are connected by hierarchical relations

**Fig. 4:**
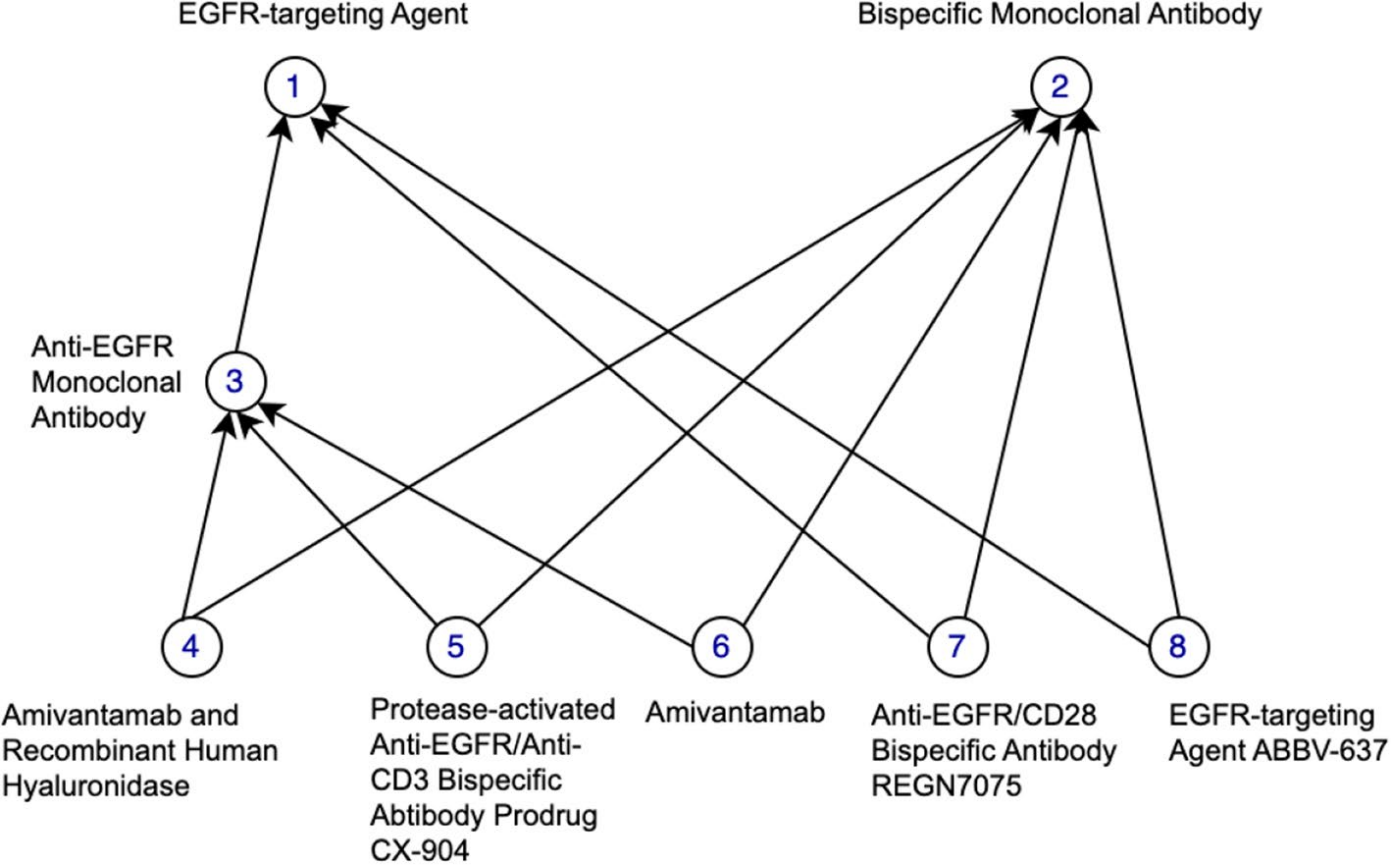
An example of non-lattice subgraphs in the 23.05e Release of NCIt. Concepts are connected by hierarchical relations

In our previous work [23, 30–32], we found that non-lattice subgraphs often reveal quality issues such as missing hierarchical relations or missing concepts. Non-lattice subgraphs are graph fragments obtained from hierarchical (or IS-A) relations of an ontology. A pair of concepts is known as a non-lattice pair if they share more than one maximal common descendant. A non-lattice subgraph can be obtained from a non-lattice pair by first reversely computing the minimal common ancestors of the maximal common descendants of the non-lattice pair and then aggregating all the concepts and hierarchical relations between them [30]. Figure 3 shows a non-lattice subgraph in the March 2020 Release of the SNOMED CT (US Edition) obtained from non-lattice pair: (“*Neoplasm of peripheral nerves of trunk (disorder)*,” “*Neoplasm of abdomen (disorder)*”) with three maximal common descendants “*Malignant neoplasm of peripheral nerve of abdomen (disorder)*,” “*Benigh ganglioneuroma of abdomen (disorder)*,” and “*Neoplasm of peripheral nerves of abdomen (disorder).*” Similarly, Fig. 4 shows a non-lattice subgraph in the 23.05e release of NCIt that contains the non-lattice pair: (“*EGFR-targeting Agent*,” “*Bispecific Monoclonal Antibody*”) and five of its maximal common descendants.

In this work, we first compute all the non-lattice subgraphs using an efficient non-lattice extraction algorithm [33]. Then we generate a list of candidate concept pairs which are concepts that are currently not linked by hierarchical relations in non-lattice subgraphs. Consider the SNOMED CT non-lattice subgraph shown in Fig. 3. Two example candidate pairs are (“*Malignant neoplasm of peripheral nerve of abdomen (disorder)*,” “*Neoplasm of peripheral nerves of abdomen (disorder)*”) and (“*Benigh ganglioneuroma of abdomen (disorder)*,” “*Neoplasm of peripheral nerves of abdomen (disorder)*”). In the NCIt non-lattice subgraph shown in Fig. 4, two example candidate pairs are (“*Amivantamab and Recombinant Human Hyaluronidase*,” “*Amivantamab*”) and (“*EGFR-targeting Agent*,” “*Bispecific Monoclonal Antibody*”).

### Logical definition-based subsumption checking

In this step, given a candidate pair, we check whether the logical definition of one concept is more general than that of the other. We perform this comparison at the relation group level. Note that some relations such as IS-A relations in Fig. 1, can be ungrouped in SNOMED CT. We consider each of these relations to be in a separate group. In addition, NCIt does not group relations as SNOMED CT does. Therefore, we also consider each relation in NCIt concepts to be in its own relation group to generalize the method’s implementation.

Based on relation groups, given a concept *X*, we consider its logical definition (inferred) as a set of groups of defining relations, $I_X=\{X_n\mid n=1, \dots , i\}$, where $X_n$ is a group of relations in the form of attribute-value pair(s), i.e., $X_n=\{(k_{nm}: v_{nm})\mid m=1, \dots , j\}$. For example, the logical definition of the SNOMED CT concept “*Neoplasm of peripheral nerves of abdomen (disorder)*” in Fig. 1 consists of three relation groups $\{X_1, X_2, X_3\}$, where $X_1=$ {(*Is a*: *Neoplasm of peripheral nerves of trunk (disorder)*)}, $X_2=$ {(*Is a*: *Neoplasm of abdomen (disorder)*)}, and $X_3=$ {(*Associated morphology*: *Neoplasm (morphologic abnormality)*), (* Finding site*: *Structure of peripheral nerve of abdomen (body structure)*)}. Note that $X_3$ contains two relations while $X_1$ and $X_2$ contain one relation each.

Given a candidate pair (*X*, *Y*), $I_X$ is considered to be more specific than $I_Y$ in logical definitions if, for each relation group $Y_m$ in $I_Y$, there exists a corresponding group $X_n$ in $I_X$ such that $X_n$ is more specific than $Y_m$. Given two relation groups, $X_n$ is considered to be more specific than $Y_m$, if for each defining relation ($k_Y$, $v_Y$) in $Y_m$, there exists a corresponding defining relation ($k_X$, $v_X$) in $X_n$ such that ($k_X$, $v_X$) is more specific than ($k_Y$, $v_Y$). The following two rules are followed to determine whether a defining relation is more specific than another.

The first rule is the inclusion rule which covers most cases. Given two defining relations ($k_X$, $v_X$) and ($k_Y$, $v_Y$), ($k_X$, $v_X$) is more specific than ($k_Y$, $v_Y$) if $k_X$ is the same as or a subtype (i.e., descendant) of $k_Y$, and $v_X$ is the same as or a subtype (i.e., descendant) of $v_Y$. Consider the candidate pair in Fig. 1. For each relation group in the inferred definition of concept “*Neoplasm of peripheral nerves of abdomen (disorder)*,” we could find a corresponding group in the inferred definition of “*Malignant neoplasm of peripheral nerve of abdomen (disorder)*” which is more specific. For example, the relation groups at the bottom of Fig. 1 both contain two relations. The relation (*Finding site*: *Structure of peripheral nerve of abdomen (body structure)*) exists under both concepts. In the other relation, the attribute type “*Associated morphology*” is the same for both the concepts while the value concept “*Malignant neoplasm of primary, secondary, or uncertain origin (morphologic abnormality)*” is a subtype of “*Neoplasm (morphologic abnormality).*” As a result, based on their logical definitions “*Malignant neoplasm of peripheral nerve of abdomen (disorder)*” is considered to be more specific than “*Neoplasm of peripheral nerves of abdomen (disorder).*”

The second rule is the property chains, which include transitive properties. Given attribute types $k_a$, $k_b$ and $k_Y$ with a property chain $k_a \circ k_b$ is a sub-property of $k_Y$, defining relation ($k_X$, $v_X$) is more specific than ($k_Y$, $v_Y$) if attribute type $k_X$ is the same as or a subtype of $k_a$, and $v_X$ has a relation to $v_Y$ via attribute type $k_b$. Consider the SNOMED CT defining relations (*Causative agent*: *Sodium calcium edetate (substance)*) from concept “*Sodium calcium edetate adverse reaction (disorder)*”and (*Causative agent*: *Edetate (substance)*) from concept “*Edetate adverse reaction (disorder)*.” Here, the value concept “*Sodium calcium edetate (substance)*” is not a subtype of “*Edetate (substance)*”. However, “*Sodium calcium edetate (substance)*” has a relation whose attribute type is “Is modification of” to “*Edetate (substance)*,” and property chain of *Causative agent* $\circ$ *Is modification of* is a sub-property of *Causative agent*. Substituting to the second rule, $k_a$ and $k_Y$ equal to “*Causative agent*,” $k_b$ equals to “*Is modification of*.” In this case, $k_X$ equals to $k_a$ (i.e., “*Causative agent*”), and value $v_X$ “*Sodium calcium edetate (substance)*” has a relation to $v_Y$ “*Edetate (substance)*” via $k_b$ “*Is modification of.*” As a result, defining relation (*Causative agent*: *Sodium calcium edetate (substance)*) is more specific than relation (*Causative agent*: *Edetate (substance)*) even though they do not comply with the first inclusion rule. In the September 2021 Release of the SNOMED CT (US Edition), all the property chains have attribute type “*Is modification of*” as intermediate property (i.e., $k_b$ = “*Is modification of*”).

In some concepts, the inferred definitions may not contain any attribute relations (only containing hierarchical relations). In such cases, we only have limited definitions for the potential supertype, and it could be meaningless to find its potential subtypes considering logical definitions. To improve the quality of suggested missing hierarchical relations, we only consider those candidate pairs where the potential supertype contains at least one attribute relation.

### Supplementary lexical-based subsumption checking

In our previous work [23, 25, 32], we found that lexical features (e.g., words and noun phrases appearing in the concept names) can be used to represent the semantic meaning of concepts. These lexical features may include information that is not conveyed through logical definitions and can be taken as supplementary features in representing the semantic meaning of concepts. In this work, we aggregate three types of lexical features from a concept name to form a lexical feature set for each concept: (1) dependency pairs of two dependencies: object of a preposition “*pobj*” and direct object “*dobj*’; (2) base noun phrases; and (3) single words that were not in dependency pairs.

**Fig. 5:**
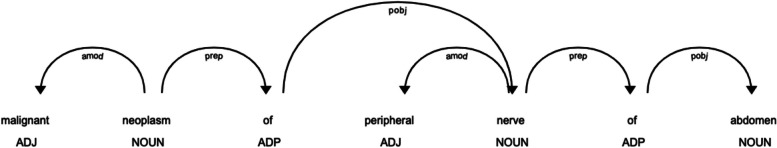
Dependency parsing result for concpet name “*Malignant neoplasm of peripheral nerve of abdomen (disorder)*.” The semantic tag “(disorder)” is not parsed and will not be included in the lexical feature set of this concept

Given a concept name, we first use Spacy [34], a Natural Language Processing (NLP) library, to perform dependency parsing. Figure 5 shows the dependency parse of the SNOMED CT concept “*Malignant neoplasm of peripheral nerve of abdomen (disorder).*” As shown, the first occurrence of the word “of” and the word “nerve” has “*pobj*” dependency. Also, the second occurrence of the word “of” and the word “abdomen” also has “*pobj*” dependency. Therefore, we include “of nerve” and “of abdomen” as dependency pairs in the lexical feature set.

Afterward, using Spacy, all the base noun phrases existing in a concept name are identified and aggregated to the lexical feature set. For instance, the SNOMED CT concept “*Malignant neoplasm of peripheral nerve of abdomen (disorder)*” contains base noun phrases: “malignant neoplasm,” “peripheral nerve,” and “abdomen”.

Finally, the rest of the words that are not part of the dependency pairs are aggregated into the lexical feature set. For instance, in the SNOMED CT concept “*Malignant neoplasm of peripheral nerve of abdomen (disorder),*” the words ‘malignant,’ ‘neoplasm,’ and ‘peripheral’ are not part of the dependency pairs “of nerve” and “of abdomen”. Therefore, these words are aggregated to the lexical feature set.

To obtain a broader view of the semantics of a concept, we further construct an enriched set of lexical features by leveraging its ancestors. The lexical features for each ancestor is computed and aggregated to the concept’s lexical feature set to generate the enriched lexical feature set. Table 1 shows the initial lexical feature set and the enriched lexical feature set for the SNOMED CT concept “*Malignant neoplasm of peripheral nerve of abdomen (disorder)*.”

**Table 1:** The initial and enriched sets of lexical features of concept “*Malignant neoplasm of peripheral nerve of abdomen (disorder)”.* Noun phrases and dependency pairs are underlined

Concept Name	Malignant neoplasm of peripheral nerve of abdomen (disorder)
Initial lexical feature set	{of nerve, of abdomen, malignant, neoplasm, peripheral, malignant neoplasm, peripheral nerve}
Enriched lexical feature set	{clinical finding, soft tissue lesion, malignant neoplasm, trunk, abdominopelvic segment, peripheral nerve disease, body, neoplasm, snomed concept, ct concept, mass, peripheral nerve, of region, of system, abdominal mass, of abdomen, of tissue, general, of nerves, body region, of trunk, nervous, peripheral nerves, nerve, peripheral nerve finding, malignant neoplastic disease, the peripheral nervous system, soft, ct, of segment, neoplastic disease, trunk structure, neoplastic, soft tissue, disorder, tumor, neurological, of structure, body site, clinical, neuropathy, malignant tumor, nervous system, abdominopelvic, peripheral, of nerve, hamartoma, neurological lesion, tissue, body system, trunk nerve lesion, malignant, abdominal, the, and/or, lesion, general finding, disease, body structure, space-occupying lesion, by site, finding}

**Fig. 6:**
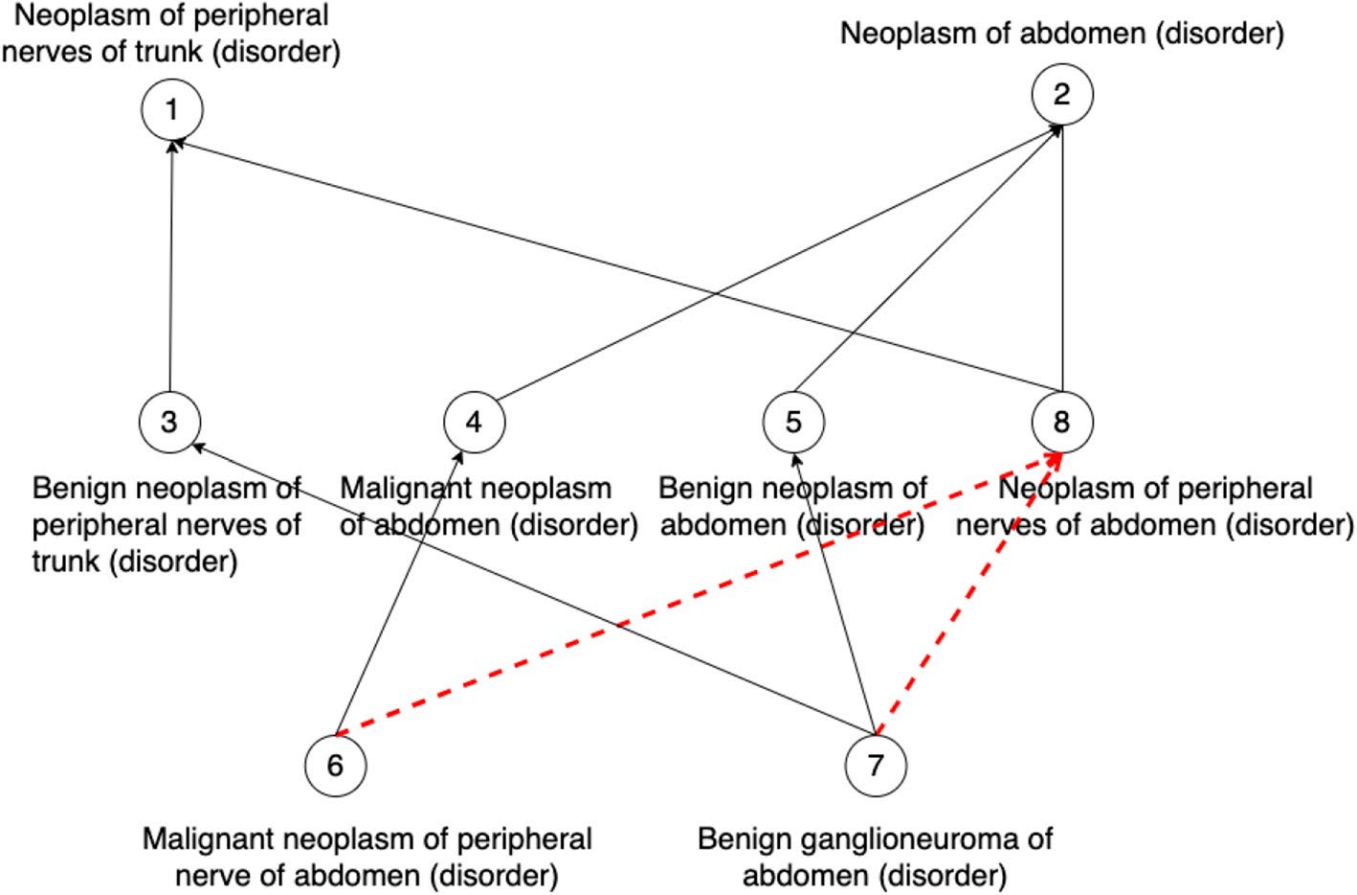
Two potentially missing hierarchical relations identified (marked red) by our methods in the SNOMED non-lattice subgraph shown in Fig. 3. Note that the original direct hierarchical relation between “*Malignant neoplasm of peripheral nerve of abdomen (disorder)*” and “*Neoplasm of peripheral nerves of trunk (disorder)*” is removed because it can now be transitively inferred by the potential missing hierarchical relation and the existing hierarchical relation

**Fig. 7:**
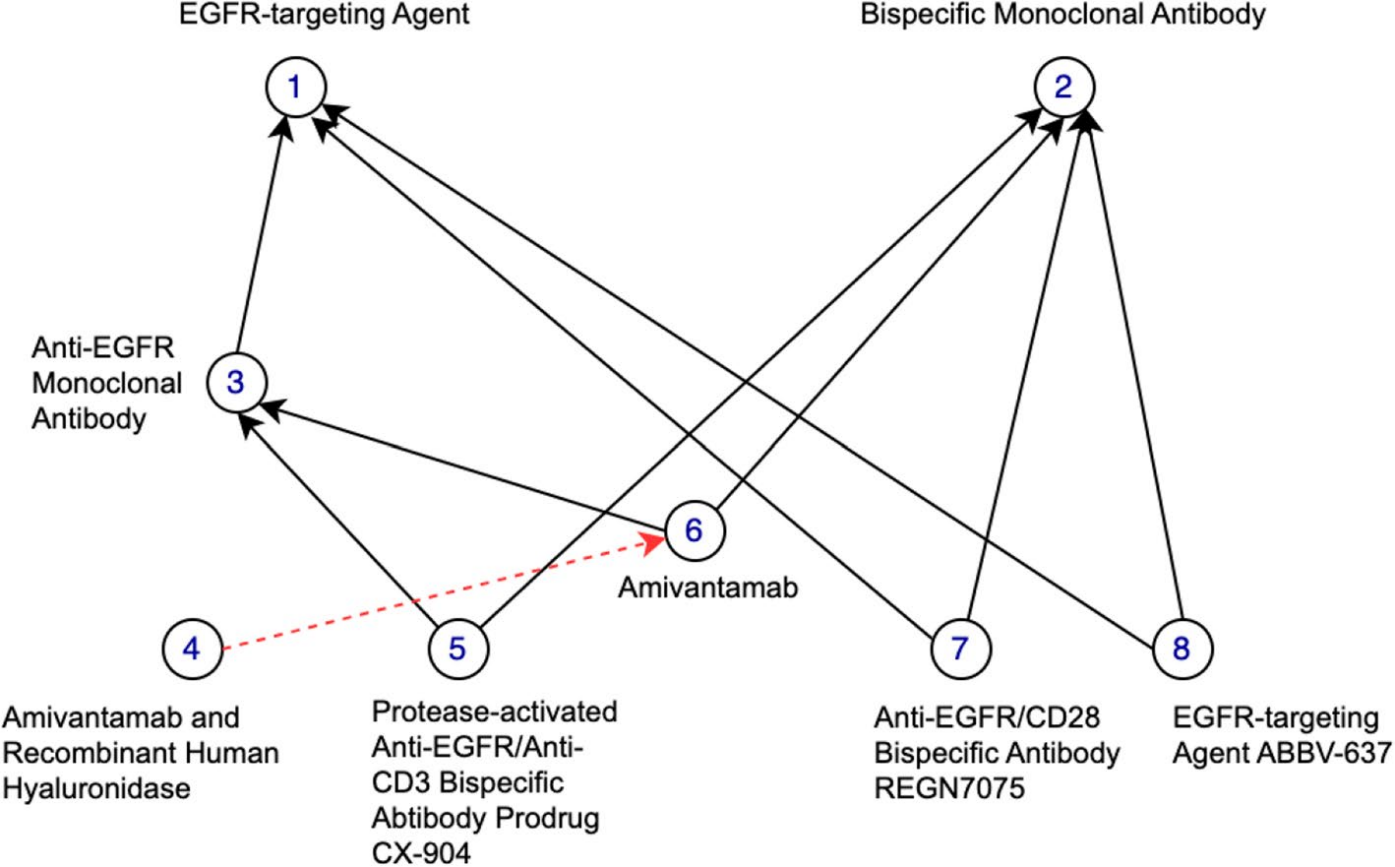
A potentially missing hierarchical relations identified (marked red) by our methods in the NCIt non-lattice subgraph shown in Fig. 4. Note that the two original direct hierarchical relations from the concept “*Amivantamab and Recombinant Human Hyaluronidase*,” to the concepts “*Bispecific Monoclonal Antibody*” and “*Anti-EGFR Monoclonal Antibody*” are removed because they can now be transitively inferred by the potential missing hierarchical relation and the existing hierarchical relations

Given a candidate pair (*X*, *Y*), if *X* is more specific than *Y* in terms of logical definitions, we further check whether the enriched lexical feature set of *X* is a superset of *Y*’s (i.e. if concept *X* is also lexical-wise more specific than *Y*). If so, a potentially missing hierarchical relation *X* IS-A *Y* is discovered. Consider the candidate pair (“*Malignant neoplasm of peripheral nerve of abdomen (disorder)*,” “*Neoplasm of peripheral nerves of abdomen (disorder)*”) in the SNOMED CT non-lattice subgraph in Fig. 3 as an example. “*Malignant neoplasm of peripheral nerve of abdomen (disorder)*” is more specific than “*Neoplasm of peripheral nerve of abdomen (disorder)*” both in logical definitions and lexical features, and therefore, a potentially missing hierarchical relation “*Malignant neoplasm of peripheral nerve of abdomen (disorder)*” IS-A “*Neoplasm of peripheral nerve of abdomen (disorder)*” is suggested by our method. Note that our approach also found another missing IS-A relation in this particular non-lattice subgraph: “*Benign ganglioneuroma of abdomen (disorder)* IS-A *Neoplasm of peripheral nerves of abdomen (disorder)*.” Both the missing IS-A relations are shown in Fig. 6. Similarly, the NCIt candidate-pair (“*Amivantamab and Recombinant Human Hyaluronidase*,” “*Amivantamab*”) in the NCIt non-lattice subgraph in Fig. 4 satisfies both these logical and lexical conditions. Therefore, a potential missing IS-A relation “*Amivantamab and Recombinant Human Hyaluronidase*” IS-A “*Amivantamab*” is suggested between these two concepts. This missing IS-A relation is shown in Fig. 7.

### Redundancy and cycle removal

Some of the potential missing IS-A suggested by our method might be implied by other potential missing IS-A relations and existing IS-A relations. For example, our approach may suggest two potentially missing hierarchical relations *A* IS-A *B* and *A* IS-A *C*. If *C* is an ancestor of *B* in the original concept hierarchy of SNOMED CT, *A* IS-A *C* will be considered redundant as it can be implied transitively by potentially missing hierarchical relation *A* IS-A *B* and existing IS-A relation *B* IS-A *C*. Such redundant potential missing IS-A relations are removed from the list of discovered potential missing IS-A relations. For each potential missing IS-A relation, we combine the rest of the potential missing IS-A relations together with all the existing IS-A relations to check whether it can be inferred.

In addition, we further remove any potential missing IS-A relations that may cause cycles in the ontology. For instance, if our method suggests two potentially missing IS-A relations *X* IS-A *Y* and *Y* IS-A *X*, then both of these would be removed as they cause a cycle. A potential missing IS-A relation could cause a cycle together with existing IS-A relations in the ontology. For example, if the method suggests *X* IS-A *Y*, while *Y* IS-A *X* already exists in the ontology, then, *X* IS-A *Y* will be removed.

### Evaluation

To evaluate the efficacy of our method in identifying accurate missing IS-A relations, we leveraged the support of domain experts (authors JS and SL) to review a sample of potential missing IS-A discovered by the method. The experts evaluated potential missing IS-A relations in terms of their validity and provided comments where necessary indicating why a certain case is valid or not. For SNOMED CT, we randomly picked potential missing IS-A relations from “*Clinical Findings*” and “*Procedure*” subhierarchies, and both the domain experts individually reviewed each case. We consider a particular potential missing IS-A relation to be valid if both reviewers agree with it. For NCIt, we picked all the potential missing IS-A relations from the “*Drug, Food, Chemical or Biomedical Material*” subhierarchy which were each manually reviewed by the author JS.

## Results

We applied our method to all the active concepts and relations in the inferred versions of the September 2021 Release of the US Edition of SNOMED CT which contained 358,356 concepts and the 23.05e release of NCIt which contained 180,065 concepts. The non-lattice detection algorithm identified 234,963 non-lattice subgraphs in SNOMED CT and 14,529 in NCIt. Among these non-lattice subgraphs, our approach identified 982 non-redundant potentially missing IS-A relations for SNOMED CT and 100 for NCIt.

### Evaluation results

From 982 potential missing IS-A relations discovered in the SNOMED CT, 577 were in the “*Clinical Finding*” subhierarchy and 247 were in the “*Procedure*” subhierarchy. For the evaluation, we randomly picked 150 potential missing IS-A relations from these two subhierarchies. The evaluation findings showed that 118 of them (78.67%) are valid IS-A relations.

Among the 100 potential missing IS-A relations detected within the NCIt, 20 were found in the “*Drug, Food, Chemical or Biomedical Material*” subhierarchy. The evaluation by the domain expert revealed that 17 of them (85%) are valid IS-A relations.

Tables 2 and 3 display five valid IS-A relations each identified within SNOMED CT and NCIt respectively.

**Table 2:** Domain expert confirmed five missing IS-A relations discovered in the SNOMED CT

Child	Parent
Folliculitis cruris pustulosa atrophicans (disorder)	Degenerative disorder of extremity (disorder)
Accidental nitrous oxide poisoning (disorder)	Accidental poisoning caused by gaseous anesthetic (disorder)
Primary squamous cell carcinoma of tonsillar pillar (disorder)	Primary squamous cell carcinoma of oropharynx (disorder)
Spinal ganglionectomy (procedure)	Ganglionectomy of peripheral nerve (procedure)
Excision of finger joint synovium (procedure)	Arthrectomy of finger (procedure)

**Table 3:** Domain expert confirmed five missing IS-A relations discovered in NCIt

Child	Parent
Palbociclib Isethionate	Palbociclib
Estramustine Phosphate Sodium Anhydrous	Estramustine
Rituximab and Hyaluronidase Human	Rituximab
Vinorelbine Tartrate Emulsion	Vinorelbine Tartrate
Liposomal Vinorelbine	Vinorelbine

## Discussion

In this paper, we introduced a method to identify IS-A relations within a terminology that were not captured during classification by Description Logic reasoners. The approach identifies unrelated concept-pairs within non-lattice subgraphs that are both logically and lexically likely to form IS-A relations.

The number of missing IS-A relations within a terminology is unknown. However, it can be assumed that the number would be much less than the number of existing IS-A relations within a well-formed terminology. Due to the discovery nature of terminology Quality Assurance, no approach is able to capture all missing IS-A relations. Different approaches usually capture different subsets of missing IS-A relations. The same approach could also capture different numbers of potential missing IS-A in different terminologies based on the characteristics of each terminology. Our approach captures a significantly higher number of potential missing IS-A relations in SNOMED CT than NCIt (982 vs 100). The major reason for this is the number of non-lattice subgraphs in each terminology. While SNOMED CT is twice the size of NCIt in terms of the number of concepts it has (358,356 versus 180,065), it contains around 16 times more non-lattice subgraphs (234,963 versus 14,529). Since our method is applied within non-lattice subgraphs, the method is able to discover many more missing IS-A relations in SNOMED CT than NCIt. It must be mentioned that any number of inconsistencies discovered is immensely valuable to the quality improvement process of these biomedical terminologies and can make a large impact on the downstream applications that use these terminologies.

**Fig. 8:**
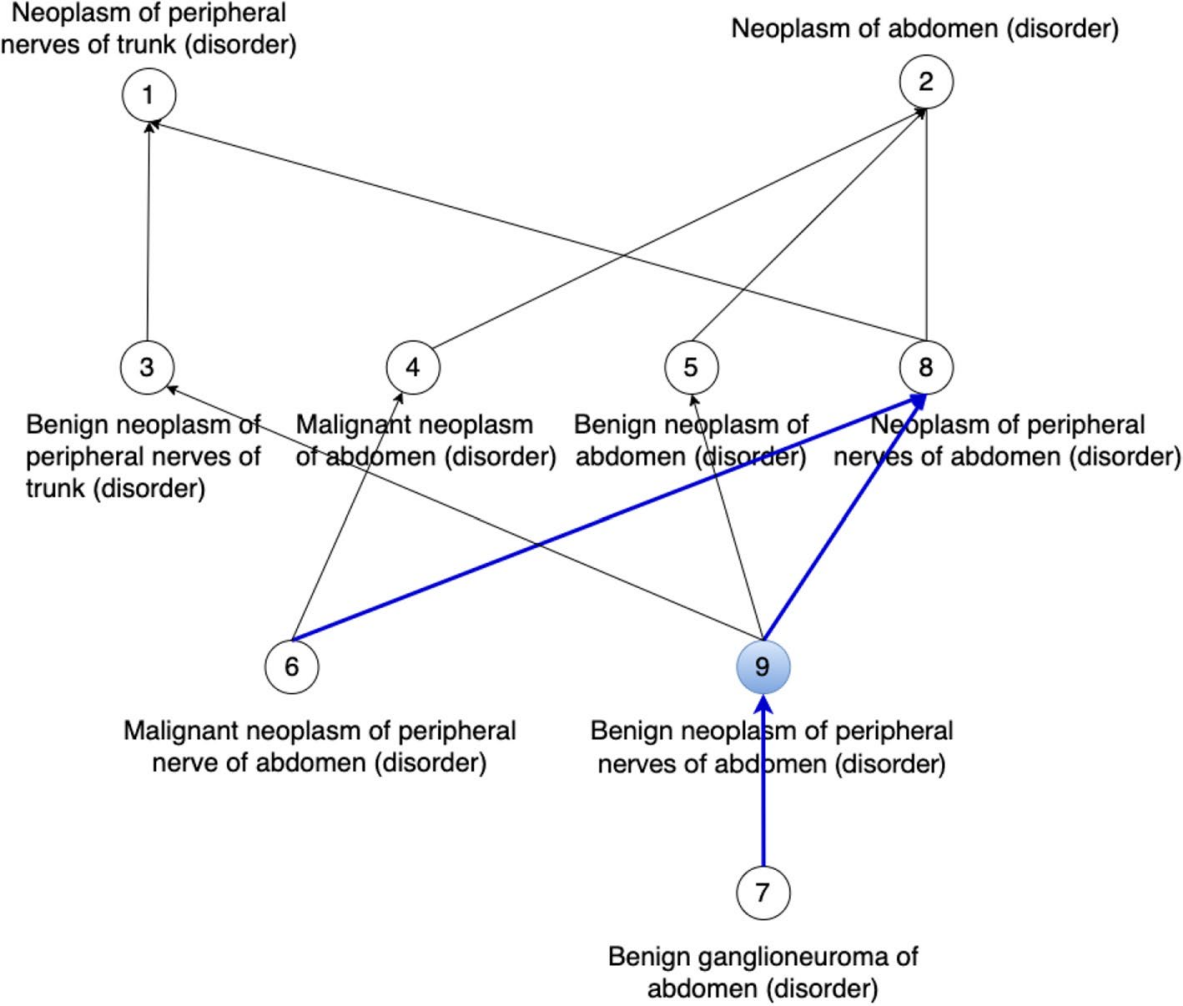
Concept hierarchy in the March 2021 Release of the SNOMED CT (US Edition) consisting of concepts in the non-lattice subgraph shown in Fig. 3. There is a new concept “*Benign neoplasm of peripheral nerves of abdomen (disorder)*” that is marked blue. The hierarchical relations marked blue are newly added ones which indicate that the missing hierarchical relations we identified were correct

Although our method uncovers missing IS-A relations between concepts, it is important to mention that rectifying such issues may not be as straightforward as directly adding the missing relations into respective terminologies as there might be other underlying issues within a terminology that cause these missing relations. For instance, the fix may rather involve modifying the logical definitions of the concepts so that the missing relation becomes inferable by a DL reasoner. For instance, Fig. 6 shows the missing IS-A relations that were suggested by our methods to the non-lattice subgraph in Fig. 3. Figure 8 presents the corresponding concept hierarchy in the March 2021 US Edition of SNOMED CT. Note that the missing IS-A relation we identified: “*Benign ganglioneuroma of abdomen (disorder)*” IS-A “*Neoplasm of peripheral nerves of abdomen (disorder)*” does not exist directly in this new version. The hierarchical relation “*Benign ganglioneuroma of abdomen (disorder)*” IS-A “*Benign neoplasm of peripheral nerves of abdomen (disorder)*” has been added in this version which together with the existing hierarchical relation “*Benign neoplasm of peripheral nerves of abdomen (disorder)*” IS-A “*Neoplasm of peripheral nerves of abdomen (disorder)*” infers the missing IS-A relation our method suggested.

It should also be mentioned that while a vast majority of missing IS-A suggestions made by our method are correct, it also makes some invalid suggestions. For example, the method suggests an IS-A relation between the SNOMED CT concepts “*Accidental fenoprofen poisoning (disorder)*” and “*Accidental poisoning caused by antirheumatic (disorder)*.” This is not correct since Fenoprofen is an NSAID medication, not an anti-rheumatic medication. The origin of this invalid suggestion stems from the fact that “*Poisoning caused by antirheumatic (disorder*)” is an ancestor of “*Accidental fenoprofen poisoning (disorder).*” This relationship affects both the logical definition-based and lexical-based subsumption checks.

Similarly, our approach suggests a missing IS-A between NCIt concepts “*Radicicol Derivative KF58333*” and “*Radicicol”.* This is also incorrect as the derivative KF58333 is a different molecule from Radicicol. This suggestion is made because the approach is only checking whether the potential child’s lexical feature set is a superset of that of the potential parent, without further looking into the semantics indicated by the additional lexical features (e.g. “derivative” in this instance) the potential child contains.

### Comparison with related work

Logical definitions and lexical features have not often been explored together for quality assurance of relations in biomedical terminologies. In one instance, Quesada-Martínez et al. have investigated natural language content in concept labels and the logical definitions to identify missing relations in SNOMED CT [35]. Their approach identifies lexical regularities from concept labels through natural language processing techniques and they propose relations between classes exhibiting these regularities. Our approach is different from this as it directly compares logical definitions across two concepts to suggest a missing relation. Quesada-Martínez et al.’s approach has identified 585 cases of potential missing relations in SNOMED CT of which they have analyzed one case which was found to be valid. Bodenreider’s approach in identifying missing hierarchical relations in SNOMED CT relies on constructing logical definitions from concept labels and running a description logic reasoner on them [12]. In contrast, our approach is applied directly to existing logical definitions of SNOMED CT and since it is applied to primitive parent terms, it captures relationships that cannot be identified through reasoning. Bodenreider’s approach has been applied to disorder and procedure concepts of SNOMED CT. The approach has uncovered 559 potential missing IS-A relations and an evaluation on a random sample with 100 cases has revealed 78% are valid. Note that the precision of our approach is slightly higher with 78.67% (118 out of 150). However, it should be mentioned that a direct comparison of precision is less appropriate to measure the effectiveness of different quality assurance approaches. This is because different approaches address different kinds of problems and may uncover distinct types of relational defects. Ontology quality assurance approaches are meant to discover ontological defects that have not been uncovered before. As there is no gold standard, it is difficult to compute recall for such approaches.

Recently, Chen et al. have introduced a deep learning-based IS-A relation prediction method for OWL ontologies [36]. Their method utilizes the pre-trained language model BERT to generate contextual embeddings for a given class with customized templates to incorporate the class context. We experimented with this approach on NCIt and found that out of the 100 potential missing IS-A relations identified by our method, 86 were also found by Chen et al.’s approach. However, it is worth noting that even when the child and parent are switched in the 100 potential missing IS-A relations, Chen et al.’s approach still predicts 69 cases as IS-A relations. Therefore, further investigations (particularly by means of a manual evaluation of the predictions) are needed for IS-A relation prediction approaches such as [36] to ensure their effectiveness in identifying missing IS-A relations.

In previous work, we have leveraged different variations of purely enriched lexical attributes to identify missing IS-A relations [23–25]. For example, in [23] and [24], the enriched lexical attributes generated were all at word-level. However, [25] introduced noun phrases in concept names to the enriched lexical attributes in addition to the words.

In [32] we investigated an approach combining enriched lexical attributes and logical definitions of concepts in NCIt to identify missing IS-A relations. However, the enriched lexical attributes generated were based on words and roots of noun chunks distinct from the method used to generate lexical attributes in this paper.

### Future directions

In this work, we obtained the enriched lexical attributes leveraging dependency-pairs, base noun phrases, and words of concepts and their ancestors. However, we did not take into account the different variations of words such as singular or plural versions as well as synonymous words and phrases. In the future, we would like to explore a comprehensive normalization strategy to normalize the lexical features leveraging lemmatization and synonym replacement approaches.

As previously stated, in certain scenarios, the underlying reason for the missing IS-A relations might be attributed to issues with logical definitions of concepts. Though we leverage logical definitions to discover missing IS-A relations in this work, we are yet unable to identify changes in logical definitions that may be needed to address the root causes. In the future, we aim to explore approaches that can tackle this important problem.

Recent advancements in Large Language Models (LLMs) have revolutionized Natural Language Processing. It would be interesting to explore how these LLMs could be effectively leveraged to make accurate predictions for missing IS-A relations.

## Conclusion

In this paper, we introduced an approach to discover missing IS-A relations that would not be captured by internal terminology consistency checking methods such as classification by description logic reasoners. Given a candidate concept-pair, our approach first compares whether the logical definition of one concept is more general than that of the other. Then, we further check whether the enriched lexical attributes of the earlier concept are a subset of the latter. If both conditions are satisfied, we suggest a potential missing IS-A relation between the two concepts. Then, we remove redundant potential missing IS-A suggestions that can be inferred and that can cause cycles. Applying our method to the September 2021 US Edition of SNOMED CT and 23.05e release of NCIt), we identified 982 and 100 potential missing IS-A relations respectively. To analyze the efficacy of our approach, an evaluation on a sample of cases was performed by domain experts. The evaluation showed that out of the 150 SNOMED CT cases, 118 are valid IS-A relations and 17 out of 20 are valid IS-A relations in NCIt. As a vast majority of cases identified by the method are accurate, this method can be deemed as an effective approach in identifying missing IS-A relations and can readily be adopted by other biomedical terminologies equipped with concept names and logical definitions.

### Supplementary Information

**Supplementary Materials 1.**

## Data Availability

Not applicable.

## Abbreviations

NCI: National Cancer Institute
NCIt: National Cancer Institute thesaurus
DL: Description logic
NLP: Natural language processing
LLMs: Large language models

## Code availability

https://github.com/rashmie/OQA_logical_definitions_lexical_features.
